# Systematic review of the outcomes of urethroplasty following urethral lengthening in transgender men

**DOI:** 10.1038/s41443-025-01132-4

**Published:** 2025-08-19

**Authors:** Paul Neuville, François-Xavier Madec, Malte W. Vetterlein, Jan Adamowicz, Łukasz Białek, Felix Campos-Juanatey, Francesco Chierigo, Andrea Cocci, Mikołaj Frankiewicz, Jakob Klemm, Guglielmo Mantica, Maciej Oszczudłowski, Elaine J. Redmond, Clemens M. Rosenbaum, Wesley Verla, Marjan Waterloos, Damien Carnicelli, Nicolas Morel-Journel

**Affiliations:** 1 Department of Urology, Hôpital Lyon Sud, Hospices Civils de Lyon, Lyon, France; 2 Claude Bernard University Lyon 1, Lyon, France; 3 Service d’Urologie, Hôpital Foch, Suresnes, France; 4 Department of Urology, University Medical Center Hamburg-Eppendorf, Hamburg, Germany; 5 Department of Regenerative Medicine, Collegium Medicum, Nicolaus Copernicus University, Bydgoszcz, Poland; 6 Department of Urology, Centre of Postgraduate Medical Education, Warsaw, Poland; 7 Andrology and Reconstructive Urology Unit, Marqués de Valdecilla University Hospital, School of Medicine, Cantabria University, IDIVAL, Santander, Spain; 8 Department of Urology, Azienda Ospedaliera Nazionale SS. Antonio e Biagio e Cesare Arrigo, Alessandria, Italy; 9 Department of Urology and Andrology, Careggi Hospital, University of Florence, Florence, Italy; 10 Department of Urology, Medical University of Gdańsk, Gdańsk, Poland; 11 Department of Surgical and Diagnostic Integrated Sciences (DISC), University of Genova, Genova, Italy; 12 Department of Urology, Cork University Hospital, Cork, Ireland; 13 Department of Urology, Asklepios Hospital Barmbek, Hamburg, Germany; 14 Department of Urology, Ghent University Hospital, Ghent, Belgium; 15 Department of Urology, AZ Maria Middelares, Ghent, Belgium

**Keywords:** Surgery, Adverse effects

## Abstract

Urethral complications following urethral lengthening in transgender men, such as strictures and fistulas, are common and frequently necessitate secondary surgical interventions. These surgeries vary significantly in their techniques and are evaluated with considerable heterogeneity, making a synthesized presentation of their outcomes valuable for guiding clinical management. This systematic review included 14 studies selected through a database search (Medline, Embase, Web of Science) that reported urethral complications after urethral lengthening. Among the 595 patients considered, 76% underwent phalloplasty and 24% underwent metoidioplasty. Our findings highlight that staged urethroplasty techniques demonstrated the lowest recurrence rates (0–25%), particularly in the management of long strictures in the pendulous urethra. In contrast, one-stage urethroplasties—especially those performed without augmentation—were associated with high recurrence rates, reaching approximately 50%, even when buccal mucosa grafts were used for augmentation. Patient-reported outcomes were documented in only one-third of the included studies, underscoring the limited functional evaluation of urethroplasty outcomes following phalloplasty. The considerable variability in urethroplasty techniques, types of genital reconstruction, and reporting standards highlights the need for more comprehensive and standardized outcome assessments. Future studies will be essential in advancing our understanding and optimizing the management of these complex cases.

## Introduction

Urethral lengthening (UL) is a possible component of total phallic construction (TPC) in transgender men [1], as the ability to void while standing is frequently identified as an important goal in masculinizing genital surgery [2]. However, UL is associated with both short and long-term complications, emphasizing the importance of lifelong follow-up for individuals who undergo this procedure [3]. These complications, which are indeed common, primarily include urethral strictures and fistulas [4], requiring additional surgeries in 30 to 50% of transgender men [5, 6] Additionally, such issues may coexist with other challenges, such as hair-bearing tissue within the urethra or the formation of diverticula, particularly in areas such as the residual vaginal cavity [7].

The surgical management of urethral complications in transgender men presents unique challenges. These arise from the absence of a corpus spongiosum to support the neourethra, the utilization of scarred or less elastic flap tissues with reduced healing capacities, and the frequent requirement for secondary surgeries to address associated complications [8]. Recent years have seen the development of treatment algorithms [9, 10] and guidelines [11] that aimed at standardizing the care of urethral strictures in transgender men, considering factors such as position or length of the stricture. However, the existing literature on this topic remains limited, particularly regarding studies that assess functional outcomes using patient-reported outcomes (PROs) [12]. Moreover, most reported techniques focus on stricture treatment, whereas the management of associated fistulas may require different therapeutic approaches [13]. In this systematic review, we aim to provide a comprehensive analysis of the outcomes of urethroplasty in transgender men, and discuss the recent treatment guidelines in light of reported clinical outcomes. This synthesis seeks to enhance understanding and inform surgical strategies for optimizing patient care in this population.

## Methods

### Search strategy

We conducted a systematic search of the Medline, Embase and Web of Science database, with the last search performed in December 2024 (Supplementary file 1). The search was restricted to studies published in English. Additional relevant literature was identified by screening references cited in a recent literature review [12] and the European Association of Urology (EAU) guidelines 2024 on urethral strictures in transgender men [11]. The review was not previously registered in any database such as Prospero.

### Eligibility and screening

After removing duplicates and records that consisted only of abstracts, the remaining articles were independently screened by two reviewers (PN, FXM) to assess eligibility. The inclusion criteria, based on the PICO framework [14], included transgender men undergoing phalloplasty or metoidioplasty for TPC. The intervention was urethroplasty, while a control group was included if reported. Outcomes were those reported in the included studies, encompassing both anatomical and functional results. Studies were excluded if they lacked detailed outcomes, focused on non-surgical management, or reported solely on cisgender populations. Any disagreements during the screening process were resolved through discussion between reviewers (PN, FXM, NMJ). A PRISMA-compliant flowchart detailing the inclusion process, from the initial database search to the final selection of studies, was created to ensure transparency and adherence to systematic review guidelines [15]. A risk of bias evaluation was performed using the JBI Critical Appraisal Checklist for Case Series [16].

### Data extraction

The following data were extracted from each included study:

1. Demographic and Procedural Information: Population size, type of TPC (e.g., phalloplasty or metoidioplasty), and underlying indication for urethroplasty (e.g., urethral stricture or fistula).
2. Surgical Techniques: Type of urethroplasty performed (e.g., augmentation urethroplasty, buccal mucosa graft (BMG), or staged procedures).
3. Outcomes: Outcomes were categorized as patient-reported outcomes (e.g., satisfaction with voiding function or the use of validated or non-validated questionnaires) and objective outcomes (recurrence rates for strictures or fistulas).
4. Follow-up: Duration of follow-up reported in months.
5. Key Findings: Summary of notable results or conclusions from each study.

### Data synthesis

The findings were synthesized and organized into a table that provided an overview of the key characteristics and outcomes for each study. Data were further grouped based on the type of urethral disease (stricture, fistula, or both) and type of reconstruction (phalloplasty, metoidioplasty, or both). The recurrence rate of complications was specifically highlighted, alongside patient-reported outcomes where available.

## Results

### Study selection

**Fig. 1:**
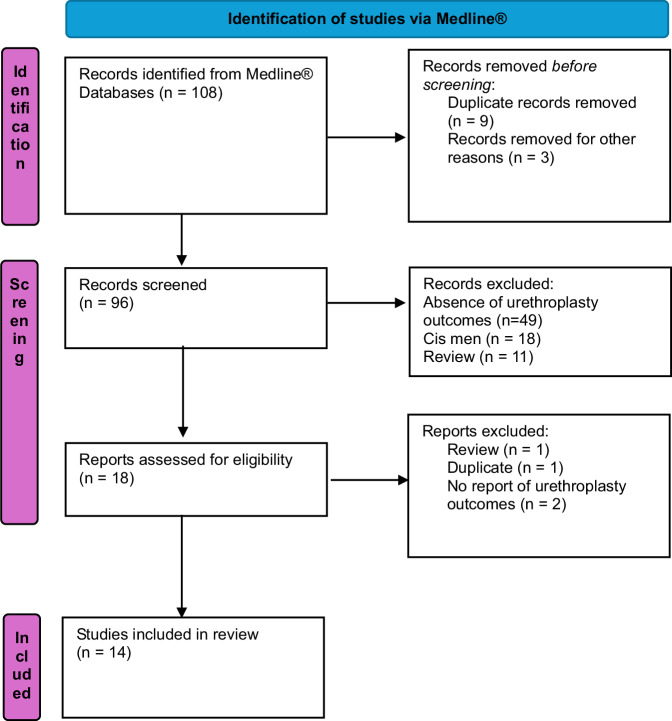
PRISMA Flow chart.

The systematic search identified 108 records from Medline®. A complementary search was conducted in the Embase and Web of Science databases, but no additional articles were identified. After automatically removing (duplicate, non-English articles), 96 records were screened. Of these, 78 articles were excluded due to irrelevance, including studies focused solely on cisgender men (*n* = 18), reviews (*n* = 11), and those without urethroplasty outcomes (*n* = 49). Eighteen reports were assessed for eligibility, and ultimately, 14 studies met the inclusion criteria and were included in the final analysis. The study selection process is detailed in the PRISMA flowchart [15] (Fig. 1).

The 14 studies included represented diverse surgical approaches, patient populations, and urethral complications. A summary of study characteristics and key findings is presented in Table 1.

**Table 1:** Qualitative synthesis of the systematic review of the literature.

Study	Procedure	Population	Type of genital reconstruction	Urethral Disease	PRO	Recurrence Rate	Mean follow-up duration (months)	Key Findings
**PHALLOPLASTY**								
Beamer et al. (2021) [18]	Single stage double-face BMGU (Group 1); Stage urethroplasty (Group 2)	14 transgender men (9 group-1; 5 group 2)	RFFF	Stricture (Prior treatment 56% in Group-1, 100% Group-2) Majority with additional complications (fistulas, vaginal remnant, additional strictures).	Mean IPSS 1 (group1) 3.9 (group2) Post-void dribbling 50%-100%	22% Group1 0% Group2	33.9 (12–60)	Staged repairs effective; single-stage feasible with healthy tissue. Treatment algorithm introduced
Lumen et al. (2011) [23]	HMP, EPA, free graft urethroplasty, pedicled flap, 2-stage	79 (76 transgender men; 3 cis men)	73 RFFF 6 ALT	118 Stricture (52 initial)	NR	41%	39	High recurrence rate for single-stage procedures; staged repairs recommended.
Paganelli et al. (2023) [7]	Meatoplasty, EPA, BMGU, skin graft urethroplasty	89 (78 transgender men; 11 cis men)	26 RFFF 19 PESP 2 ALT 1 latissimus dorsi	Stricture (*n* = 48) and other phalloplasty associated complications	LUTS score 8.4 + /− 4.9	30%	66 ( + /− 44)	High complication rates regardless of reconstruction type.
Pariser et al. (2015) [17]	1-stage BMGU	10 patients (9 transgender men; 1 cis men)	RFFF	Stricture	NR	50%	9.5 (2.7–84)	BMGU may be more effective than endoscopic management, but failure remains common
Reddy et al. (2023) [24]	HMP, EPA, 1-stage Johansen urethroplasty	71 transgender men	39 RFFF 29 ALT 2 latissimus dorsi 1 other	Stricture	NR	52% Overall 58% after EPA 25% after 1-stage urethroplasty	30	Staged urethroplasty has the lowest failure rate among urethroplasties
Rohrmann et Jakse (2003) [19]	EPA, BMGU, 2-stage urethroplasty	25 transgender men	RFFF	14 Strictures and fistulas	NR	28%	NR	Pedicle skin graft is the best option for fistulas associated with short stricture
Schardein et al. (2020) [21]	Double-face BMGU	8 transgender men	RFFF	Stricture	Mean IPSS 3.1 (0–11), IPSS QoL 0.9 (0–3)	25%	31 (10–56)	High patient satisfaction with upright voiding restoration.
Schardein et al. (2022) [20]	Staged BMGU for long pendulous strictures Redo vaginectomy (7/17)	17 transgender men	15 RFFF 2 ALT	Stricture > 7 cm	Improved markedly in 11/13 (85%), moderately in 2/13 (15%)	12%	24 (4–77)	Staged approaches effective for strictures >7 cm with high patient satisfaction.
Verla et al. (2019) [22]	EPA urethroplasty	44 transgender men	35 RFFF; 9 ALT	Short isolated stricture ≤3 cm after DVIU failure	NR	43%	40 (7–125)	Stricture length and extravasation at first voiding are predictors of failure.
Wilson et al. (2016) [27]	Fasciocutaneous flap reinforcement of BMGU	3 patients (2 transgender men; 1 cis men)	RFFF	Stricture, 2 fistula	All voiding while standing	33%	7-43	Fasciocutaneous reinforcement reduces tension, improves outcomes.
**METOIDIOPLASTY**								
Lumen et al. (2020) [26]	Fistuloplasty, ventral meatotomy, HMP, 2-stage, pedicled flap	26 transgender men	Metoidioplasty	14 fistula 8 stricture 4 both	NR	33% after urethroplasty 39% after fistuloplasty	15	Fistuloplasty and urethroplasty are associated with failure in one-third of patients.
De Rooij et al. (2022) [25]	Open urethroplasty (HMP, BMGU, fistulectomy, redo vaginectomy)	96 transgender men	Metoidioplasty	31 Stricture 44 Fistula 21 Both	NR	18% after open urethroplasty for urethral stricture 28% after open urethroplasty for fistula	36 (14–123)	Open techniques superior to endoscopic methods; colpectomy improves outcomes.
**BOTH PHALLOPLASTY AND METOIDIOPLASTY**								
De Rooij et al. (2022) [10]	HMP, EPA, 2-stage with or without graft, graft, pedicled flap, DVIU, Dilation	72 transgender men	56 Phalloplasty 16 Metoidioplasty	147 Sticture (78 initial, 69 recurrent)	NR	37% (43% after phalloplasty, 24% after metoidioplasty)	61 (25–202)	Highest success rates were seen after HMP in short strictures; and after graft, pedicled flap, or 2 stage urethroplasties in longer or more complicated strictures. Higher success rates after metoidioplasty vs phalloplasty
Jung et al. (2023) [40]	HMP, BMGU, 2-stage Johansen urethroplasty	41 transgender men	36 Phalloplasty 5 Metoidioplasty	Stricture (46% were located at the distal pars fixa including the pars fixa / pars pendulum anastomosis)	NR	8% for BMGU 66% for HMP 25% for 2-stage Johansen	30 (12–52)	Substitution urethroplasty optimal for mid-length strictures; staged for longer strictures.

*BMGU* Buccal mucosa graft urethroplasty, *HMP* Heineke-Mikulicz procedure, *EPA* Excision primary anastomosis, *DVIU* Direct vision internal urethrotomy, *NR* Not reported, *PRO* Patient Reported Outcome, *RFFF* Radial forearm free flap, *ALT* Anterolateral thigh flap, *PESP* pre-expanded supra-pubic flap.

### Study characteristics

The included studies involved a total of 595 patients undergoing urethroplasty, primarily following phalloplasty (*n* = 452, 76.0%, most commonly utilizing a radial forearm free flap) or metoidioplasty (*n* = 143, 24.0%). The majority of participants were transgender men, although a few studies also included cisgender men, albeit rarely (2.7%). Urethral strictures were the most frequently reported complications, with many studies also addressing fistulas and combined urethral issues. Follow-up durations varied significantly, ranging from 4 to 202 months, with the majority of studies reporting a mean follow-up period exceeding two years. The risk of bias analysis is reported in Table 2 [16]. A notable source of uncertainty across all included studies was the reliability of outcome measurements, particularly in assessing recurrence, which was largely defined by the absence of reintervention. This assessment may have been impacted by loss to follow-up, a limitation that is especially relevant in retrospective studies.

**Table 2:**
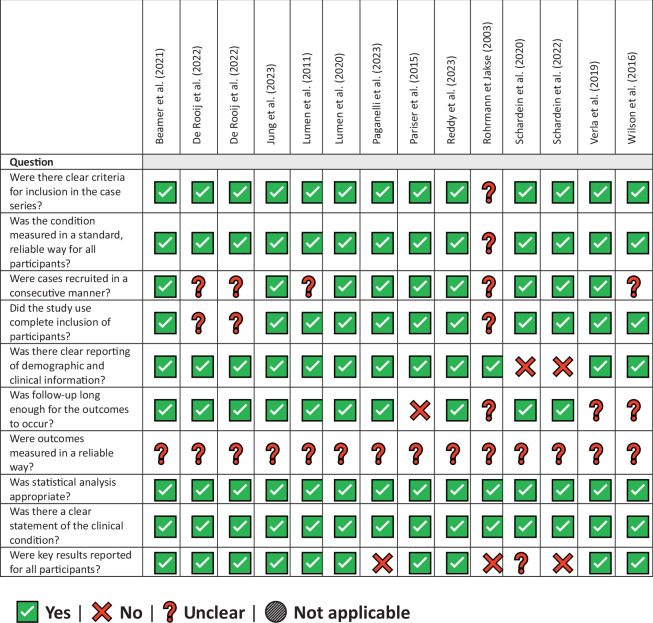
Risk of bias evaluation: JBI critical appraisal checklist for case series.

### Surgical approaches and outcomes

**Fig. 2:**
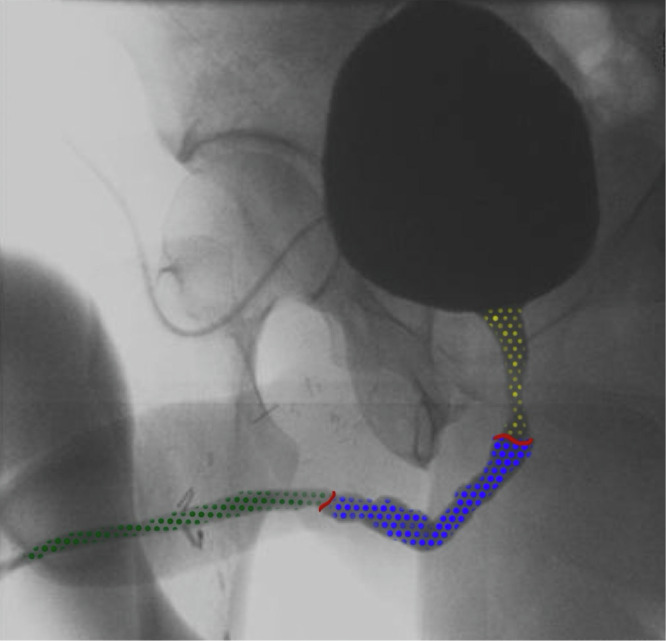
Urethral lengthening anatomy. Green dots: pars pendulans urethra. Blue dots: pars fixa urethra. Yellow dots: native urethra. Red lines: proximal urethral anastomosis and distal urethra anastomosis.

**Fig. 3:**
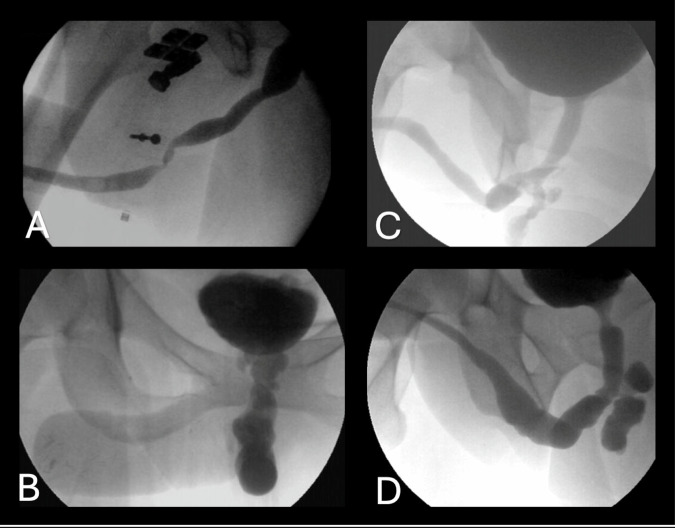
Examples of urethral complications. **A** Stricture at the distal urethra anastomosis between pars fixa and pars pendulans. **B** Complete obliterative stricture with retrograde dilation of the pars fixa. **C** Urethral fistula with the perineum. **D** Urethral fistula with a vaginal diverticulum.

To provide a clearer understanding of the outcomes from various studies, Fig. 2 presents an example of UL anatomy following phalloplasty, while Fig. 3 illustrates examples of urethral complications. Several urethroplasty techniques were employed across studies, including single-stage [9, 17, 18] and staged repairs [19, 20], buccal mucosa graft urethroplasty (BMGU) [7, 17, 21], excision and primary anastomosis (EPA) [22], pedicled flaps [23], Heineke-Mikulicz procedure (HMP) [23–25] and ventral meatotomy [26].

While one-stage repairs offer the advantage of fewer surgeries, they were associated with higher recurrence rates, particularly in cases involving poor tissue quality or extensive scar tissue [18, 20, 23, 24].

Staged urethroplasty consistently demonstrated lower recurrence rates, particularly in managing complex or long strictures, as shown in studies by Beamer et al. [18] and Schardein et al. [20], both of which utilized BMG for staged augmentation. Success rates (i.e., absence of recurrence) for staged procedures ranged from 100% to 75%, these procedures were often employed for longer and/or more complex strictures.

BMGU was effective for mid-length strictures but demonstrated recurrence rates as high as 50% in some cases [17].

In the only study comparing outcomes between the two techniques, success rates of urethroplasty were higher following metoidioplasty compared to phalloplasty [10]. Conversely, the highest recurrence rates were observed after urethroplasty performed on phalloplasty.

The techniques used for fistula repair were not described in detail, except for the mention of fistulectomy, which could be combined with more complex procedures such as redo-vaginectomy—though again, specific details of the surgical methods were not provided.

### Patient-reported outcomes

PROs were included in only five studies [7, 18, 20, 21, 27], underscoring a notable gap in the functional evaluation of outcomes. Furthermore, the recurrence rate was consistently the sole criterion used for anatomical evaluation, indicating a need for broader and more comprehensive assessment criteria. High levels of satisfaction were reported with staged repairs and double-face BMGU [20, 21], particularly regarding restoration of upright voiding.

## Discussion

In this systematic review, we analyzed a total of 585 urethroplasty procedures performed primarily in transmen, mostly following phalloplasty, with a smaller number occurring after metoidioplasty. In rare instances, outcomes of urethroplasty in cisgender men were included in the reported data. The urethra in phalloplasty is often constructed using a combination of local mucosal flaps for the pars fixa and skin flaps or grafts for the pars pendulans, creating a structure composed of different types with varying supportive tissues and healing capacities. The urethra in metoidioplasty is often constructed using a combination of local mucosal flaps, with adjunction of BMG or vaginal mucosa graft; this approach closely resembles the construction of the pars fixa in phalloplasty. The main complications arise from the distal urethral anastomosis and the pars pendulans, where skin is commonly used. In contrast, BMG is the preferred graft material for redo urethroplasty [28].

Our findings underscore that staged urethroplasty techniques demonstrated the lowest recurrence rates, with this approach predominantly reported for managing long strictures in the pendulous urethra. Conversely, one-stage urethroplasty, especially when performed without augmentation (e.g., HMP or end-to-end anastomosis), was associated with high recurrence rates, reaching approximately 50%. Even with augmentation BMGU, recurrence rates remained notable and significantly higher than those reported for urethroplasty in cisgender patients.

PROs were reported in only a third of the included studies, highlighting the lack of a functional evaluation in urethroplasty outcomes after phalloplasty. The PROs varied across the five studies that incorporated them, ranging from the ability to void while standing to questionnaires assessing urinary symptoms or perceived improvement after surgery, using a mix of ad-hoc and validated questionnaires. This variability made it challenging to synthesize the findings in a cohesive manner. The definition of success in urethroplasty and the standardized criteria for its evaluation remain subjects of ongoing debate, notably in the context of cisgender men [29, 30]. However, there is consensus that a comprehensive assessment should encompass both anatomical and functional outcomes [31–33]. Sole reliance on recurrence rates offers a limited perspective on anatomical success [29], particularly in retrospective studies, which comprised the entirety of the studies included in this review. Future studies are encouraged to report both functional and anatomical success using more robust and standardized criteria, as suggested by various guidelines on urethroplasty in cisgender men [28, 33, 34].

The EAU guidelines on urethral stricture have included a dedicated section for transgender men since their initial publication [11]. In the latest version, the recommendations are limited to delaying urethroplasty for at least six months following phalloplasty, and favoring staged urethroplasty for strictures in the neophallus urethra. This preference is supported by consistently high patency rates observed across studies using staged technique. However, the lack of robust evidence in the available studies limits the recommendation to a “weak” strength.

One-stage, non-augmented procedures such as HMP or EPA are mentioned as options, primarily for strictures located at the distal urethra anastomosis (pars fixa–pars pendulans anastomosis) [28]. Despite this, these techniques are associated with high recurrence rates and are not included as formal recommendations. Their use may be limited to short (<1.5 cm), non-complex strictures, as outlined in a recent disease management algorithm [10].

Our review has several limitations, the most significant being the risk of bias across the included studies. Notably, all the studies were retrospective in nature, lacking predefined protocols or prespecified outcomes of interest. Additionally, missing data were not consistently reported or adequately explained, which can have a considerable impact, especially in retrospective studies with relatively small sample sizes. Another limitation lies in the inherent complexity of phalloplasty reconstruction, which introduces substantial variability in the anatomy of the reconstructed urethra [35]. Additional variability can arise from the inclusion of peritoneal grafts [36] reinforcement with a pedicled gracilis flap [37], or the impact of an associated colpectomy [38], further contributing to the complexity of the reconstructed urethra. While this variability is less pronounced, it is still present in cisgender men, where differences in supporting tissue and vascularization are observed between the penile, bulbar, and prostatic urethra. Differences may allowpr for alternative techniques for urethroplasty, as urethral closure under suprapubic tunnel or abdominal pedicled skin flap which have recently been described in cismen [39]. The predominance of transgender men in the studies did not allow for comparison despite anatomical differences. Conversely, urethroplasty outcomes clearly differ between metoidioplasty and phalloplasty, as highlighted in the study by De Rooij et al. [10] These differences underscore the importance of reporting outcomes for these procedures separately to ensure accurate and meaningful comparisons. Another source of variability in phalloplasty reconstruction lies in the urethral environment. Factors such as vaginal remnants or mucoceles, hair-bearing urethra, and the presence of urinary stones are relatively common in some cohorts [7, 18] but are infrequently reported across studies. These factors introduce significant potential for confounding bias in the analysis of urethroplasty outcomes, further complicating the interpretation of results.

Further studies are encouraged to improve the analysis of outcomes following urethroplasty after phalloplasty, as a more comprehensive understanding is crucial for advancing patient care. In this context, it is important that outcomes are reported and analyzed with attention to key factors such as stricture length, location, the type of tissue comprising the urethra at the stricture site, associated complications, and the urethroplasty techniques utilized. As highlighted earlier, the assessment of outcomes should include both functional and anatomical evaluations to accurately measure the success of this complex procedure. A multi-institutional study is currently being developed by the YAU–EAU Reconstructive Group (Phalloplasty-Associated Neourethral Treatment for Strictures, PANTS Study), and aim to provide a more comprehensive understanding of this complex procedure.

## Conclusion

This review highlights the complexity of urethroplasty after phalloplasty, with staged techniques showing lower recurrence rates compared to one-stage procedures. Variability in reconstruction methods, tissue composition, and reporting standards underscores the need for more comprehensive and standardized outcome analyses. Future studies, such as the ongoing PANTS Study by the YAU–EAU Reconstructive Group, are essential to improve our understanding and management of these challenging cases.

## Supplementary information

Supplementary file 1
