# Supplementary material for: Systematic review of the outcomes of urethroplasty following urethral lengthening in transgender men
Source: Int J Impot Res. 2025 Aug 19;38(4):302–10. doi: 10.1038/s41443-025-01132-4 (PMC13132719; doi:10.1038/s41443-025-01132-4)
Supplement: Supplementary file 1 [file 41443_2025_1132_MOESM1_ESM.doc]

Search line

`[(urethroplasty) AND ((phalloplasty) OR (metoidioplasty) OR (transgender)]`.
